# Intralesional EBV-DNA load as marker of prognosis for nasopharyngeal cancer

**DOI:** 10.1038/s41598-019-51767-9

**Published:** 2019-10-28

**Authors:** Johan S. Nilsson, Ola Forslund, Fredrik C. Andersson, Malin Lindstedt, Lennart Greiff

**Affiliations:** 1 Department of ORL, Head & Neck Surgery, Skåne University Hospital, Lund, Sweden; 2 Department of Clinical Sciences, Lund University, Lund, Sweden; 3 Department of Laboratory Medicine, Medical Microbiology, Lund University, Lund, Sweden; 4 Department of Laboratory Medicine, Clinical Genetics and Pathology, Skåne University Hospital, Lund, Sweden; 5 Department of Immunotechnology, Lund University, Lund, Sweden

**Keywords:** Head and neck cancer, Tumour virus infections

## Abstract

Nasopharyngeal cancer (NPC) is associated with the Epstein-Barr virus (EBV). The clinical presentation and prognosis of NPC is well described, but not in relation to intralesional EBV-DNA load. In a retrospective design, 48 patients with NPC were examined. Patient history was re-evaluated, and diagnostic biopsies were re-examined. Furthermore, intralesional EBV-DNA was quantitated and HPV status determined. Cancer stage, disease-free survival (DFS), and overall survival (OS) were assessed. Of the 48 patients, 36 (75%) patients featured lesions that were positive for EBER (Epstein–Barr virus-encoded small RNA) and 40 (83%) were positive for EBV-DNA. Seven patients (15%) were HPV positive. The levels of EBV-DNA ranged from 0.0005 to 94617 copies/cell. An EBV-DNA load of more than 70 copies/cell was associated with a prolonged DFS for EBV-DNA positive patients treated with curative intent (p = 0.046). In conclusion, the EBV-DNA load in NPC lesions appears to vary greatly. For patients with EBV-DNA positive NPC treated with curative intent, an EBV-DNA load of more than 70 copies/cell is associated with a better outcome in terms of 7-year DFS.

## Introduction

Nasopharyngeal cancer (NPC) is a squamous cell carcinoma (SCC) that arises in the epithelial lining of the nasopharynx^1^. Its geographical prevalence is highly diverse, being uncommon in most parts of the world, with age-adjusted incidences of less than one per 100,000, but much more common in specific areas, e.g. Hong Kong (21.4 per 100,000 among males)^2^. The incidence of NPC in Sweden is 0.2–0.5 cases per 100,000 inhabitants, which has been consistent at least since the 1970s (the Swedish National Board of Health and Welfare; www.socialstyrelsen.se/statistik; 1970–2017).

Epstein-Barr virus (EBV), identified by Epstein *et al*. in 1964^3^, is linked to several malignancies including NPC, as was first reported in the early 1970s^4,5^, and presence of EBV-DNA in NPC-lesions was explored in Sweden already in 1979^6^. NPC is associated with EBV in more than 50% of cases worldwide, but in regions such as southern China this figure is more than 95%^7^. Primary EBV infections, with up to 90% of the world population being infected^8^, may not solely explain the geographical differences. Instead, the aetiology of NPC is considered to be multi-factorial and to include also a genetic susceptibility as well as dietary and social risk factors^1,9^.

EBV-DNA in plasma can be employed to monitor disease activity in NPC. For example, it has been used successfully to screen for NPC and to detect early tumour stages^10^ as well as to follow-up treatment effects^11^. Furthermore, plasma EBV-DNA has been suggested as a mean to stratify treatment of NPC^12,13^ and to determine prognosis, where high levels have been associated with a poor prognosis^14^. In contrast, EBV positive NPC-lesions, often indicated by a presence of EBERs (Epstein–Barr virus-encoded small RNAs), are associated with a favourable prognosis, in comparison with EBV negative cases^1^.

Data on intratumoral EBV-DNA have focused on its specificity and sensitivity in relation to a diagnosis of NPC^15–17^. Similarly, presence of EBV-DNA in brush samples from NPC lesions has been suggested as a clinical tool for NPC^18–20^. However, clinical characteristics in relation to actual EBV-DNA load in NPC seem to be lacking. This type of information may be valuable, as indirectly suggested by reports on oropharyngeal cancer (OPC) and human papilloma virus (HPV)-DNA, where clinical characteristics have been associated with the degree of viral load, *e*.*g*., improved overall and disease-free survival in patients with the highest viral loads, and presence of regional metastases associated with low viral load^21,22^.

HPV has been described in NPC-lesions, and a small series of studies have examined clinical characteristics in relation to HPV status^23–27^. HPV positive NPC has been reported to feature poor local control and survival, while distant failures may be less common (in contrast to EBV-associated disease)^23,25^. On the other hand, in a recent study involving 956 patients, HPV neither correlated with nor predicted survival^27^. Also, a meta-analysis could not definitely conclude whether or not HPV-positivity correlated to overall survival (OS)^26^. However, Ruuskanen *et al*. recently reported that HPV positive NPC was correlated to increased survival^24^. Arguably, HPV-status is needed as background information when analysing virus-associated NPC.

In this study, primary NPC biopsy material from patients with untreated disease was reviewed and analysed. In addition to histological characterization, a particular aim was to quantitate the intralesional EBV-DNA load and HPV-status. The findings were correlated to clinical characteristics of NPC including cancer stage according to TNM, disease free survival (DFS), and OS.

## Materials and Methods

### Study design

The study was of a retrospective design and involved an analysis of 48 patients with NPC diagnosed between 2001 and 2015. Patient data was re-evaluated and tumour biopsies were re-examined focusing on histology and EBER expression. Also, EBV-DNA was quantitated, and presence of high-risk HPV examined. Cancer stage, DFS, and OS were assessed. All methods were performed in accordance with the relevant guidelines and regulations.

### Patients and ethical approval

Data on consecutive patients with previously untreated SCC of the nasopharynx managed at the Departments of ORL, Head & Neck Surgery and Oncology, Skåne University Hospital, Lund, Sweden, a tertiary referral centre with Southern Sweden as catchment area, between 2005–2015, were retrieved (n = 98). Of these cases, a formalin-fixated paraffin-embedded (FFPE) primary biopsy material from the cancer lesions was available for 48 individuals. Among these 48 cases, positive *in situ* hybridization for EBER (EBER-ISH), where the original slides were still available, was present in 16 cases. FFPE tissue from the remaining 32 cases, where EBER-ISH either had not been performed (n = 27), or been negative (n = 2), or of insufficient quality (n = 2), or unavailable for review (n = 1), were retrieved from the archives.

Patient records were reviewed and all cases were re-assessed, and re-classified if indicated, according to the TNM classification system (7^th^ edition)^28^. One patient was lost to follow-up due to emigration. Accordingly, 47 patients were available for outcome analysis. All patients were treated with curative intention except for nine who were diagnosed with stage IVC disease and one with locally advanced intracranial extension.

Ethical approval was granted by the Regional Ethical Review Board at Lund University (2014/117). In accordance with the ethical approval, informed consent was not required due to this being a historical biopsy material. The study design was advertised in selected printed media prior to effectuation, with the possibility to opt-out, as specified in the approval.

### Histopathologic review

Retrieved FFPE tissue, was sectioned and slides prepared. All histological slides were reviewed and classified according to the World Health Organization (WHO) Classification of Head and Neck Tumours (2017 edition)^29^. EBER-ISH for EBER1 and EBER2 were performed, for cases where this information was not already available, on four µm thick sections using an INFORM EBER Probe with ISH iVIEW Blue Detection Kit on BenchMark Ultra (all Ventana Medical Systems, Tucson, AZ). Appropriate positive specimen controls were used as well as Negative Control Probe (Ventana Medical Systems), the latter for assessment of non-specific background staining. The pathologist (F.C.A.) was unaware of the outcome of EBV-DNA analyses, while performing the classification. The methodology used for EBER analysis was the same as used for the historic cases. It has previously been demonstrated that EBERs are stable in formalin-fixed paraffin-embedded tissues^30^, and no difficulties were experienced with the present analysis.

### EBV-DNA quantification

Sections of FFPE blocks were prepared. In-between each case-block, a paraffin blank-block was sectioned as a control for contamination. For each case, new gloves and a new knife was used. The blank-block was sectioned first. Four 5 μm sections were transferred to a 1.5 mL screw-cap Eppendorf tube using a sterile instrument. From all sections, blank-blocks, and case-blocks, DNA was extracted with an automated xylene-free method using a purification kit (ES-S110FP-C, ExScale, Biospecimen Solutions, Uppsala, Sweden) in an automated system (Magtration System magLEAD 12GC, ExScale Biospecimen Solutions), and was then eluted in 100 μL elution solution.

From each sample 10 μL was analysed for quantity of EBV DNA (single copy gene encoding the Epstein-Barr virus Nuclear Antigen 1 (EBNA1)) by the use a commercial kit (EBV/ISIN/100, GeneProof, Brno, CZ) for real time PCR (ABI7500). Quantification was extrapolated from a linear regression standard curve obtained from four standards included in the kit containing 5 × 10^6^ to 5 × 10^3^ copies EBNA1 per mL. Since sample size varied, the number of cells per sample were calculated by quantification of the Beta-globin gene, which was amplified with PC03 and PC04 primers in a 25 μL PCR reaction containing 2.5 μL template^31^.

The standard curve was obtained from serial dilutions of 5 × 10^4^ to 50 copies per PCR of the beta-globin gene (D7011, Sigma-Aldrich, Stockholm, Sweden). For the calculations of number of human cells per sample, the copy number of beta-globin was divided by two. We assumed that each human cell carries two beta-globin gene copies and that a diploid genome equivalent contains ~6.6 pg DNA. Water samples were included as negative controls. The number of EBV DNA copies/cell in each sample was calculated as follows: number of EBV DNA copies per μL/number of cells per μL.

### HPV typing and p16 analysis

As described previously^32,33^, the DNA-extractions from the samples were analysed for presence of 40 different HPV subtypes using a multiplex Modified General Primers (MGP)-PCR and Luminex. In addition, HPV positive lesions were also subjected to immunostaining for p16. Four µm sections of FFPE tissue were prepared utilising the CINtec p16-kit, Clone E6H4, (Ventana Medical Systems), and the BenchMark Ultra platform (Ventana Medical Systems). Positive p16 expression was defined as at least 70% nuclear and cytoplasmic expression with moderate to strong intensity^34^.

### Statistics

χ^2^-tests were performed to explore associations between gender and NPC-specific death, between tumour-stage and gender, EBER status as well as EBV-DNA status, respectively, and between HPV and EBER status as well as EBV-DNA status, respectively. Mann-Whitney U-tests or Kruskal-Wallis tests (as appropriate) were used to analyze EBV-DNA load in relation to gender and tumour stage. Mann-Whitney U-tests were used to analyze age in comparison to death (non-specific and NPC-specific). DFS, NPC-specific survival, and OS (in relation to EBV-DNA load with various cut-offs, age, gender, tobacco use, and tumour stage I-II *vs*. III-IV and I-III *vs*. IV, respectively) were described by Kaplan-Meyer curves and significance levels were determined by log-rank tests. When appropriate, Cox regression analyses were performed for confounder identification.

### Compliance with ethical standards

This article does not contain any studies with human participants or animals performed by any of the authors. This is a retrospective study and it was performed in accordance with the ethical standards of the institutional and/or national research committee (the Ethics Committee at Lund University, reference number: 2014/117) and with the 1964 Helsinki declaration and its later amendments or comparable ethical standards. Information to potential study participants still alive was provided according to specific instructions (advertisement) from the Ethics Committee at Lund University.

## Results

### Characteristics

Patient characteristics are presented in Table 1. Briefly, 15 out of 48 (31%) patients were diagnosed with T1 lesions, 41 (85%) were N-positive, and 9 (19%) featured distant metastases (M-positive). Female patients were diagnosed at more advanced stages than male patients (p < 0.022, χ^2^-test). The shortest follow-up time for patients still alive was 3.5 years, the median follow-up period was 6.4 years, and 20 patients had a follow-up of more than seven years. The 5- and 7-year OS was 75% and 65%, respectively. Neglecting deaths, 40 and 31 patients, respectively, were eligible for 5- and 7-year follow-up.

**Table 1:** Patient characteristics.

	Total	Male	Female
Number	48	34	14
Age (min/median/max)	22/57/80	22/57/80	26/53/79
Tobacco (1/2/3/4)*	13/13/19/3	10/8/13/3	3/5/6/0
T1/T2/T3/T4**	15/18/3/12	12/12/3/7	3/6/0/5
N0/N1/N2/N3**	7/18/17/6	6/14/12/2	1/4/5/4
M0/M1**	39/9	29/5	10/4
Stage I/II/III/IV**	4/13/9/22	4/11/7/12	0/2/2/10

*1. Daily smoker; 2. Ex-smoker (>1 years); 3. Never-smoker; 4. Missing information. **Tumor-Node-Metastasis classification of malignant tumors (2017 edition) of the Union for International Cancer Control^28^.

### Histopathology

After histopathological re-examination, 36 out of 48 (75%) patients presented EBER positive lesions. All of these were non-keratinizing undifferentiated cancer. Thus, the remaining twelve were EBER negative. Out of these, five were keratinizing SCC and six non-keratinizing undifferentiated cancer. The remainder was SCC without further specification. No cases of non-keratinizing differentiated cancer or basaloid SCC were thus identified.

### EBV-DNA load

**Figure 1:**
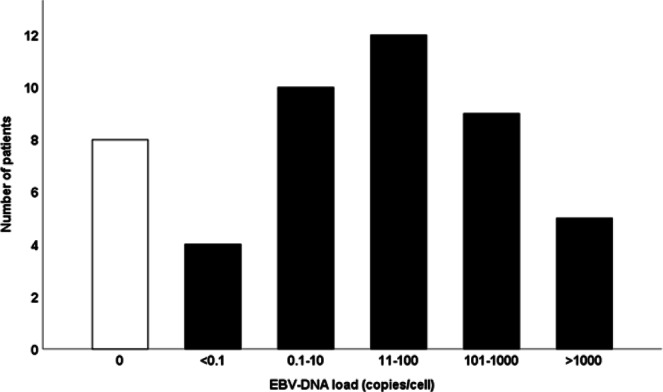
Number of subjects per EBV-DNA load group.

Forty out of 48 (83%) patients presented EBV-DNA positive lesions. These cases comprised all the 36 EBER positive and four additional EBER negative ones. Quantification of EBV-DNA showed a large span ranging from 0.0005 to 94617 copies/cell (Fig. 1). Gender was not correlated to EBV-DNA load (Mann-Whitney U-test). The four cases with the lowest virus load (<0.1 copies/cell) were identical to the four EBER negative cases.

### Cancer stage

Neither EBV-DNA status (χ^2^-test), EBER status (χ^2^-test), nor EBV-DNA load (Kruskal-Wallis test) did differ between cancer stages. Similarly, between *Stage I* + *II* and *Stage III* + *IV*, i.e. the cut-off point for introduction of chemotherapy in most protocols, there were no differences associated with EBV-DNA status (χ^2^-test), EBER status (χ^2^-test), or EBV-DNA load (Mann-Whitney U-test).

For advanced cancer (stage IV) in comparison to stages I-III, a trend towards stage IV being associated with EBV negative cases was observed (EBV-DNA status p = 0.070, EBER status p = 0.094, χ^2^-test). This trend emerged as significant when HPV positive cases were omitted (see below). For EBV-DNA load no such trend was seen (Mann-Whitney U-test). Distant metastases at diagnosis were not present in any case of EBV-DNA load >1000 copies/cell (n = 5).

### Disease-free survival

**Figure 2:**
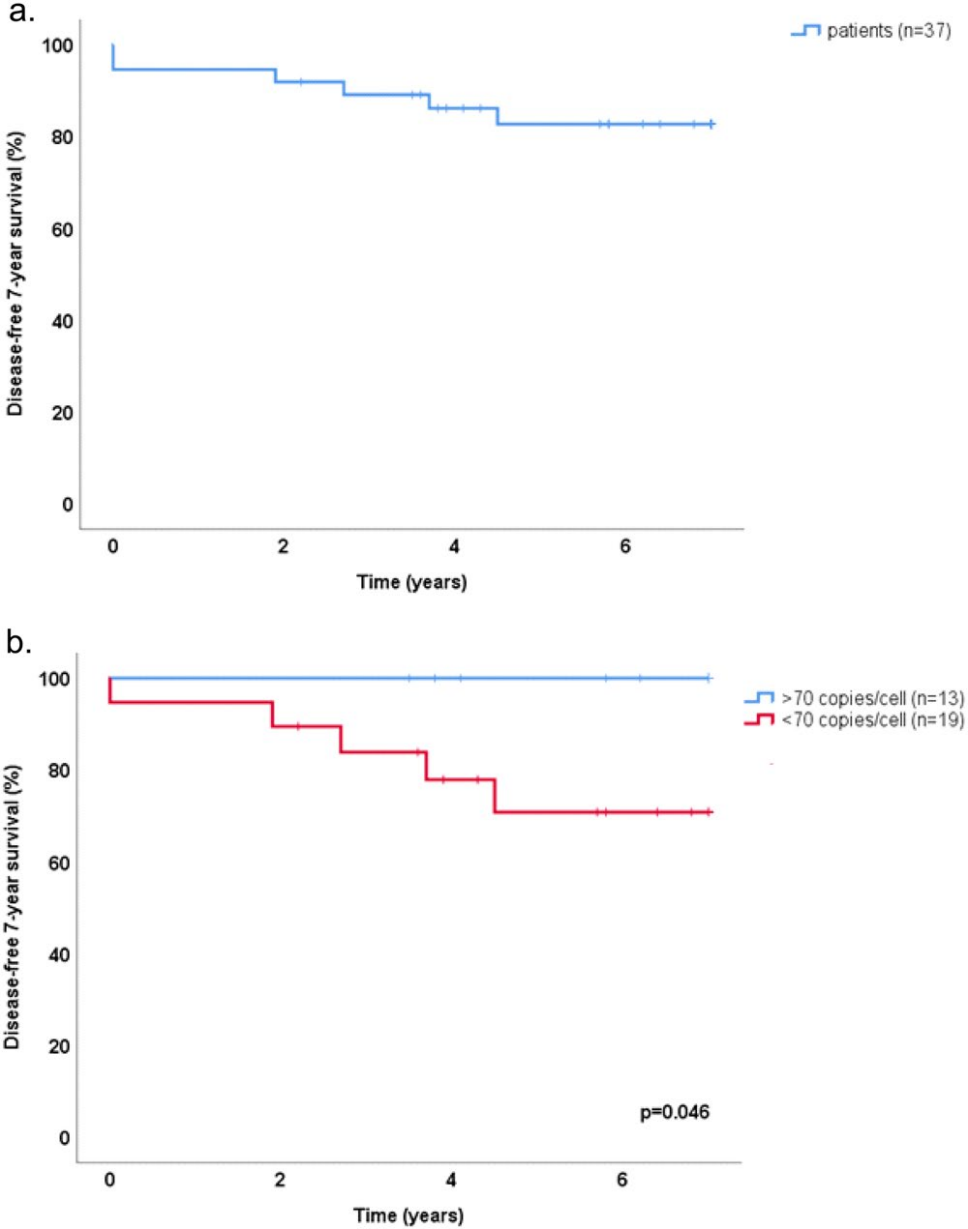
Kaplan-Meier estimates of 7-year DFS for the study population as a whole (**a**) and for EBV-DNA positive NPC cases grouped according to EBV-DNA cell copy number (**b**). Vertical lines mark events (residual or recurrent cancer) and crosses mark end of follow-up before 7 years. (DFS: disease free survival; NPC: nasopharyngeal cancer).

DFS was defined as time from diagnosis to either end of follow-up (seven years or death) or identification of residual or recurrent disease. DFS for the study material as a whole is shown in Fig. 2a. Thirty-seven patients treated with curative intent were eligible for analysis, omitting ten who were treated with non-curative intent and one who was lost to follow-up. Overall there were only seven specific events, comprised of two residual tumours and five recurrences. There was no difference in DFS correlated to gender (log-rank test).

When omitting EBV-DNA negative cases (n = 8), a significant difference in DFS was observed between low and high EBV-DNA load with a split of the material at 70 copies/cell (p = 0.046, log-rank test) (Fig. 2b). When the analysis was repeated omitting all EBER negative cases (n = 11), statistical significance was still reached (p = 0.050, log rank test) (data not shown). Due to no events among cases with >70 copies/cell the material was not deemed eligible for regression analysis. However, log-rank tests for tobacco use and tumour stage were performed, and failed to show statistical significance.

### Overall survival

**Figure 3:**
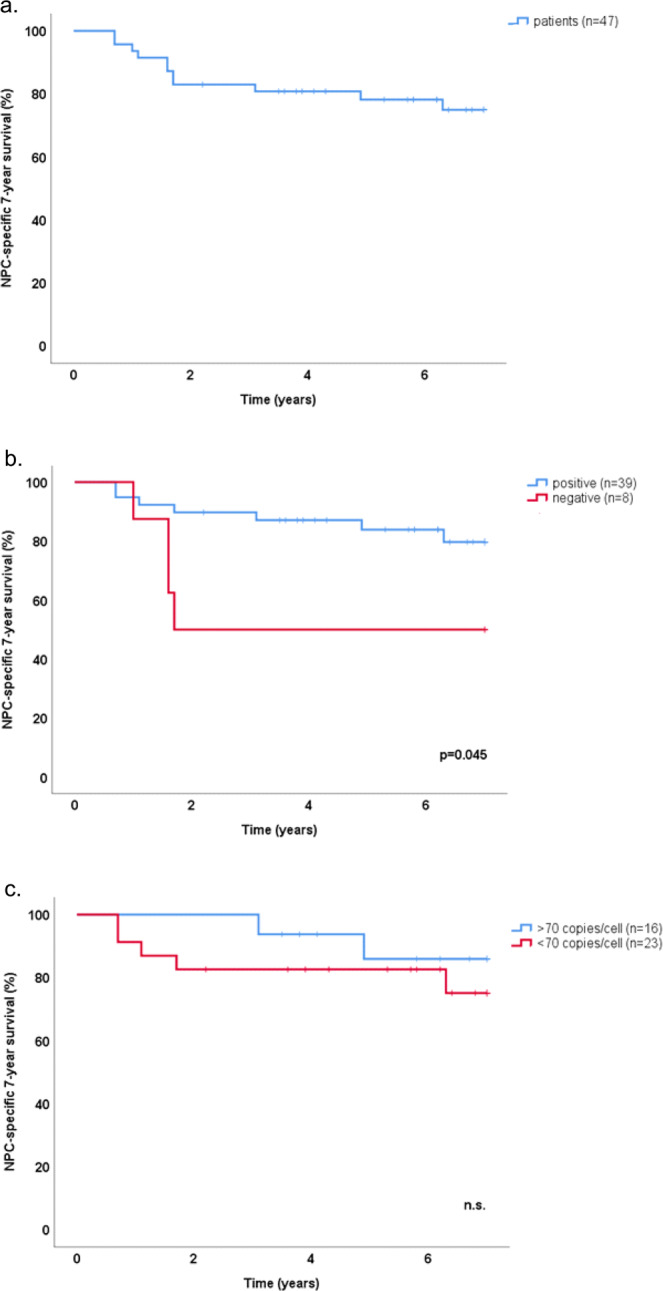
Kaplan-Meier estimates of 7-year NPC-specific survival for the study population as a whole (**a**), grouped according to EBV-DNA status (**b**), and for EBV-DNA positive cases grouped according to EBV-DNA cell copy number (**c**). Vertical lines mark events (NPC-specific death) and crosses mark end of follow-up before 7 years. (NPC: nasopharyngeal cancer).

Overall 13 deaths occurred during the follow-up period, at 3.5–7 years, and eleven of these were NPC-specific. NPC-specific 7-year survival is shown in Fig. 3a. Ten out of eleven NPC-specific deaths occurred among patients presenting in stage IV (n = 21, p = 0.00043, χ^2^-test). There was significant association between age at diagnosis, and overall death (p = 0.042), but not with NCP-specific death (Mann-Whitney U-test). There was no gender-association for NPC-specific death, neither for stadium IV were there was a female predominance as described in Table 1 above (χ^2^-test), nor in the group as a whole (log-rank test).

NPC-specific 7-year survival with the material split according to qualitative EBV-DNA status (positive *vs*. negative) showed a significantly worse outcome among negative patients (p = 0.045, log-rank-test) (Fig. 3b), an outcome that was not reproduced with EBER-status even if a trend was observed (p = 0.095, log-rank-test). Regarding EBV-DNA status, a follow-up bivariate Cox regression analysis showed positive M-status as being a much stronger predictor for NPC-specific death (p = 0.00059). NPC-specific 7-year survival with the material split at EBV-DNA 70 copies/cell is shown in Fig. 3c.

### HPV

**Figure 4:**
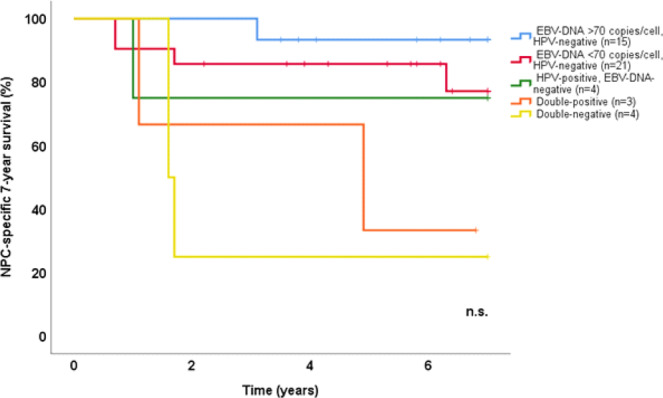
Kaplan-Meier estimates of 7-year NPC-specific survival according to viral status. Vertical lines mark events (NPC-specific death) and crosses mark end of follow-up before 7 years. (NPC: nasopharyngeal cancer).

Seven of the 48 tumours (15%) were positive for HPV, apportioned to HPV16 in four cases, and HPV18, HPV45, and HPV56 in one case each. Thus, all these lesions featured high-risk HPV types^35^. Five were T2 lesions and two were T4-lesions. Three were EBV-DNA positive, with EBV-DNA loads of 184, 0.0041 and 0.0010 copies/cell, of which the latter two were also EBER negative. Five of the HPV positive cases were also positive for p16. These were all EBER negative, and consisted of three EBV-DNA negative cases, and two cases with very low EBV-DNA load (0.0041 and 0.0010 copies/cell). Overall, the presence of HPV was highly associated with negative EBV-DNA (p = 0.0019, χ^2^-test) as well as with negative EBER (p = 0.000060, χ^2^-test). The NPC-specific 7-year survival with regards to combined viral presence is shown in Fig. 4. When omitting HPV positive cases (n = 7), advanced NPC (stage IV in comparison to stage I-III) was significantly correlated to negative EBV-DNA (p = 0.017, χ^2^-test, as well as negative EBER (p = 0.035, χ^2^-test), but not for EBV-DNA load (Mann-Whitney U-test).

## Discussion

In this study, we demonstrate a large variation in the EBV-DNA load of NPC lesions. Furthermore, that an EBV-DNA load of more than 70 copies/cell is associated with prolonged DFS for patients with EBV-DNA positive NPC lesions treated with curative intention. Taken together, our observations suggest the possibility that the EBV-DNA load in NPC has clinical implications.

EBER is considered the gold standard for EBV’s association to NPC, which incidentally is based on data from screening and not from demonstrated disease^17^. EBV-DNA was present in 83% of the NPC lesions in this study. While the association between EBER positivity and EBV-DNA was strong, it was not absolute: 75% of the NPC lesions that were EBV-DNA positive were also EBER positive. Furthermore, the remaining four EBV-DNA positive (and EBER negative) cases were those with the lowest DNA load. In the present report, data are presented with and without EBER negative cases included into the EBV positive group (see Results section), but our illustrations only show data for EBV-DNA positive cases regardless of EBER status. Our findings suggest that intralesional EBV-DNA load may be a potential marker of EBV-driven disease, but its importance in comparison to EBER needs to be examined in future studies.

The EBV-DNA load varied markedly, from 0.0005 to 94617 copies/cell, which to the best of our knowledge is the second observation in this field of its kind. Accordingly, Shao *et al*., in a material of 49 patients with endemic EBER positive NPC, reported a median EBV-DNA load of 27.8 copies/actin (quartiles 6.35–279.5)^36^. Similarly, Faust *et al*.^22^, focusing on quantitative HPV-DNA in OPC, a disease frequently associated with HPV, reported on a wide range of HPV-DNA in tumour lesions: from 0.003 to 1079 copies/cell. Arguably, our observation (this study) and those by Shao *et al*.^36^ and Faust *et al*.^22^, suggest the possibility that the molecular milieu within a cancer lesion (NPC or OPC), e.g., the degree of immunological activity, may vary depending on the viral status and have implication on disease prognosis etc. In agreement, in OPC, a presence and function of intratumoral HPV-specific T cells correlates to a greater chance to respond to standard treatment^37^. Also, regulatory CD8+ T cells and CD4+ Th17/Th1 subsets are present at higher levels among tumor-infiltrating HPV16-specific T cells in HPV-driven OPC (*c*.*f*. OPC negative disease)^37^. A clear link between high (*c*.*f*. low) viral load and immunological activity is to our knowledge not yet demonstrated for either OPC or NPC.

It is suggested that the clinical presentation of NPC may depend on the viral status of the cancer lesion, i.e., it being EBV negative or positive, but findings are diverging. For example, Stenmark *et al*.^25^ found no correlation between viral status and tumour stage, while Ruuskanen *et al*.^24^ demonstrated that EBV positive tumours present with a lower T-stage than EBV negative tumours. In contrast, for the present study material as a whole, no difference in cancer stage was observed between patients with EBV negative and EBV positive disease. However, focusing on a subgroup of patients with HPV negative NPC, advanced disease (stage IV) was associated with EBV negative cancer lesions. On the contrary, again in this study, EBV-DNA load was not associated with clinical stage of disease at the time of presentation. Taken together, available data suggest that EBV may not be a major determinant of cancer stage at presentation in NPC.

In this study, we verified previous observations that EBV negative NPC features a poor prognosis compared with EBV positive disease^1,25^. Adding to these observations, this study provides data on the implication of EBV-DNA load. Thus, EBV-DNA load of more than 70 copies/cell, a cut-off level chosen after explorations into various levels and their relation to outcome (i.e., DFS, OS, and NPC-specific survival), was associated with improved DFS for EBV-DNA positive patients treated with curative intention. This association also remained when omitting EBER negative EBV-DNA positive cases. Furthermore, patients with a very high EBV-DNA load, more than 1000 copies/cell (1237–94617 copies/cell, n = 5) did not feature distant metastases at diagnosis, neither residual nor recurrent tumours (follow-up 5.8–12.3 years after diagnosis). In contrast to Shao *et al*.^36^, our study relates EBV-DNA load to outcome. Taken together, our observations underscore the notion that the prognosis of NPC may vary due to viral status and further add that also viral DNA load may be of importance in that context. Our observations are similar to those reported by Faust *et al*.^22^ where low amount of HPV16-DNA was associated with lymph node metastases among OPC-patients.

The present finding that EBV positive NPC lesions, and particularly such featuring high levels of EBV-DNA, feature a favourable prognosis (i.e., greater DFS) does not contradict observations that high levels of EBV-DNA in plasma, or in “extralesional” brush samples at diagnosis, are associated with more advanced NPC and poor prognosis^18–20,38,39^. Arguably, while EBV-DNA within the NPC lesion may reflect an inert capacity of the tumour that predicts a certain course, EBV-DNA in plasma may indicate whether or not an EBV positive NPC has been successfully treated (a reduction in EBV-DNA levels) or has recurred following successful treatment (an increase in EBV-DNA levels). Further studies are warranted to elucidate the utility of EBV-DNA in NPC lesions in relation to EBV-DNA in plasma.

A particular concern focusing on intralesional EBV-DNA is that the result may depend on the size of the biopsy, the relative presence of cancer cells in the tissue (i.e., the heterogeneity of the lesion), the possible presence of EBV-DNA in non-tumour cells, and somatic genetic aberrations in the cancer cells. With regard to biopsy size, EBV-DNA was normalized against the beta-globin gene, whereas the relative presence of cancer cells and other cells, hypothetically including also lymphocytes that may be chronically infected by EBV, is an unknown factor in the present analysis. As regards the possibility of somatic genetic aberrations affecting the copy number of *HBB*, the gene that encodes beta-globin, NPC is generally diploid and there are no reports of recurrent gains or deletions involving this locus^40,41^. Taken together the concerns addressed should not affect our overall results/conclusions, particularly given the logarithmic scale range of EBV-DNA revealed by the present study.

Although the focus for NPC is on EBV, a sub-group of these patients may feature HPV positive disease^15,23–25,27,42–47^. In agreement with recently reported data from a Finnish NPC cohort^24^, 15% of the tumours examined in this study were HPV positive, which is, as expected, less than for OPC but more than for other head and neck cancers. Moreover, even if the presence of EBV and HPV have been deemed almost mutually exclusive in NPC^25,48^, a supposition which may be supported by the above mentioned Finnish study that demonstrated no “double-positive” tumours among 150 NPC-cases^24^, three “double-positive” cases were observed in the present study. However two of these were EBER negative and also featured very low levels of EBV-DNA. The present p16 analysis (an acknowledged surrogate marker of HPV-driven disease), which was positive in 5/7 of the HPV-DNA positive cases, may support HPV as the principle viral driver in these lesions. However, one must consider that NPC associated with EBV may also be p16 positive^49^.

It may be speculated that a viral aetiology is favourable to a non-viral one, because the virus-induced oncogenic changes, in contrast to mutation-driven ones, feature a less aggressive cancer phenotype. In accordance, Chua *et al*. have suggested that “*HPV negative/EBV positive NPC*” more than “*HPV positive/EBV negative NPC*” more than “*non-viral associated NPC*” predict a favourable outcome^1^. This way of sub-classifying NPC was further elaborated in this study. Although the sub-groups groups were too small to allow for a statistical analysis, the patterns visualised (Fig. 4) warrant further studies within the field, e.g., viral status in relation to treatment differentiation.

We conclude that the EBV-DNA load in NPC lesions varies greatly and that a high load is associated with a better outcome in terms of 7-year DFS. Furthermore, our observations suggest the possibility that HPV should also be evaluated in NPC.

## Data Availability

The datasets generated during and/or analysed during the current study are available from the corresponding author on reasonable request.
